# Urine proteins reveal distinct coagulation and complement cascades underlying acute versus chronic lupus nephritis

**DOI:** 10.1172/JCI186143

**Published:** 2025-10-01

**Authors:** Ting Zhang, Jessica Castillo, Anto Sam Crosslee Louis Sam Titus, Kamala Vanarsa, Vedant Sharma, Sohan Kureti, Ramesh Saxena, Chandra Mohan

**Affiliations:** 1 Division of Rheumatology, the Second Affiliated Hospital, Zhejiang University School of Medicine, Hangzhou, Zhejiang, China.; 2 Department of Biomedical Engineering, University of Houston, Houston, Texas, USA.; 3 Department of Internal Medicine, University of Texas Southwestern Medical Center, Dallas, Texas, USA.

**Keywords:** Autoimmunity, Immunology, Coagulation, Complement, Lupus

## Abstract

The current gold standard for assessing renal pathology in lupus nephritis (LN) is invasive and cannot be serially repeated. To assess if urine can serve as a liquid biopsy for underlying renal pathology, urine obtained from patients with LN at the time of renal biopsy were interrogated for 1,317 proteins, using an aptamer-based proteomic screen. Levels of 57 urine proteins were significantly elevated and correlated with pathology activity index (AI), notably endocapillary hypercellularity, fibrinoid necrosis, and cellular crescents. These included proteins pertaining to leukocyte/podocyte activation, neutrophil activation, endothelial activation, and markers of inflammation/anti-inflammation. In contrast, complement and coagulation cascade proteins, and proteins related to the extracellular matrix (ECM) emerged as the strongest urinary readouts of concurrent renal pathology chonicity index (CI), notably tubular atrophy and interstitial fibrosis. In vitro mechanistic studies revealed that complement proteins C3a and C5a increased the expression of profibrotic ECM proteins in macrophages and proximal tubule epithelial cells. Thus, carefully assembled panels of urinary proteins that are indicative of high renal pathology AI and/or CI may help monitor the status of renal pathology after therapy in patients with LN, in a noninvasive manner, without the need for repeat renal biopsies.

## Abstract 2

Lymphoid, Myeloid and Vascular cell activation underscore renal pathology activity in lupus nephritis, while complement and coagulation co-activation mark chronicity – implications for a liquid biopsy

## Introduction

Lupus nephritis (LN) occurs in about 50% of patients with systemic lupus erythematosus (SLE) and is a major cause of morbidity, which substantially increases the risk of kidney failure and death (1, 2). LN is categorized into 6 classes by the International Society of Nephrology and Renal Pathology Society (3). Classes of proliferative LN (III, IV, or III/IV + V) are most severe, carrying the highest risk of progression to end-stage kidney disease, which occurs in 10% to 30% of patients with LN. Death directly attributable to kidney disease occurs in 5% to 25% of patients with proliferative LN within 5 years of onset (2). In addition to LN classes, kidney lesions are also appraised using the NIH activity and chronicity indices (3), which may guide treatment and predict long-term outcome. High AI (AI) and CI values are independent predictors of end-stage kidney disease (4). Thus, accurate evaluation of kidney lesions, and timely and aggressive management are essential to improve prognosis in LN.

Kidney biopsy is the gold standard for the characterization of LN and is currently the only definitive approach to assess the degree of renal inflammation and damage in LN. Although the role of a biopsy at first presentation of kidney involvement in lupus is well established, the significance of a repeat kidney biopsy is increasingly emerging. Kidney function impairment is predicted by both AI and CI and by some of their components at repeat biopsy, but not at first biopsy (5). Moreover, protocol repeat biopsies have shown considerable discrepancies between clinical and histological findings (2). Despite apparent clinical response to immunosuppressive therapy, repeat biopsies have revealed persisting active nephritis in almost half of the patients (6), and those with increased NIH AI had a disease flare over the course of 24 months (7). Chronic renal damage increased after induction therapy even in complete clinical responders. Conversely, patients who have complete histological remission may still be clinically active (8). Thus, repeat renal biopsies may provide invaluable guidance and help identify patients who could benefit from intensified treatment (9).

Renal biopsy is invasive and inconvenient, however, and associated with potential complications such as bleeding and infection (10). Moreover, the reading of a renal biopsy by pathologists is subjective with substantial inter-pathologist discordance (11). Thus, noninvasive biomarkers precisely predicting histological activity and chronicity are urgently needed. With the advent of proteomics, several urine biomarkers of LN have been reported, providing an alternative noninvasive approach to monitor renal histology (12, 13). Among these, only a handful of biomarkers correlate well with LN histological activity or chronicity, such as VCAM-1 (14), CD163 (15), L-selectin (16), and angiostatin (17). To undertake a more comprehensive screen to uncover urinary proteins predicting renal pathology AI and CI, urine samples procured at the time of biopsy from patients with LN with different histological AI and CI were subjected to unbiased proteomics in this study.

## Results

### Proteins and pathways implicated in urine samples of patients with LN with high AI.

**Figure 1:**
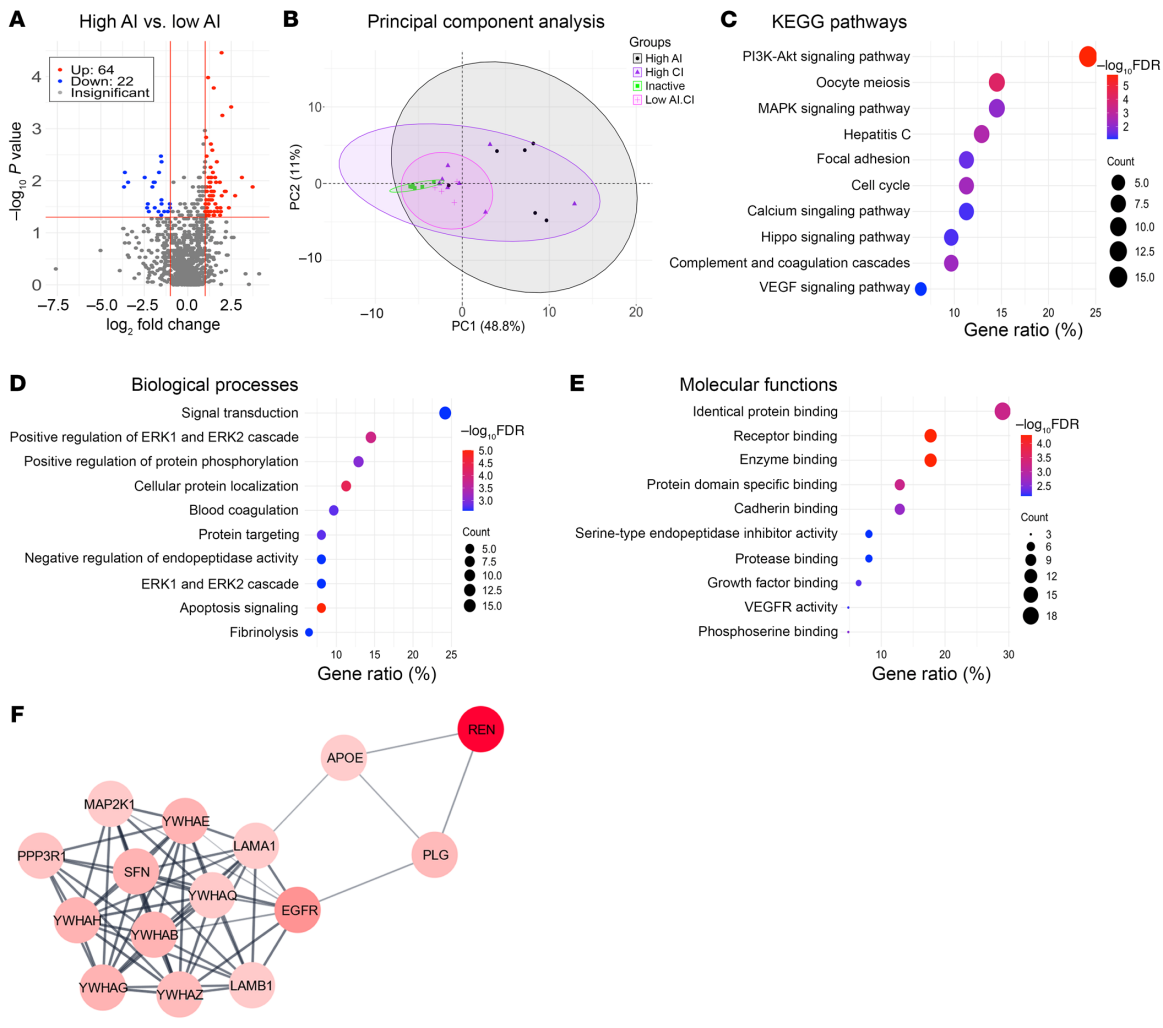
Proteins elevated in urine samples of patients with LN with high renal pathology AI. (**A**) A volcano plot representation of all 1,317 proteins assayed in the aptamer-based screen. Log-transformed data were used for the analysis. In total, 64 proteins were identified as being significantly upregulated with an FC of greater than 2 and a *P* value of less than 0.05 (red dots). An additional 22 proteins were identified as being significantly downregulated, with a FC of less than 0.5 and a *P* value of less than 0.05 (blue dots). Among the 64 upregulated proteins, 57 proteins (high-AI proteins, or Hi.AI in the figure) had the strongest Spearman correlation with renal pathology AI (*r* > 0.6); all these proteins also had ROC AUC values of greater than 0.775 when comparing patients with LN with Hi.AI with patients with LN with non-Hi.AI. (**B**) A PCA plot of the 57 significantly elevated proteins in participants with Hi.AI (FC > 2; *P* < 0.05; Spearman’s *r* > 0.6 with AI). The principal components (PCs) are displayed on each axis of the plot. Concentration ellipses encompass each subject group, color coded as indicated. (**C**–**E**) The 57 proteins from the aptamer-based screen whose levels were elevated in participants with Hi.AI with a FC of greater than 2, a *P* value of less than 0.05, and a Spearman’s *r* of greater than 0.6 with AI were used for GO and KEGG pathway enrichment analyses. The implicated top 10 KEGG pathways (**C**), biological processes (**D**), and molecular functions (**E**) identified using the Database for Annotation, Visualization and Integrated Discovery (DAVID) are displayed. Each annotation was identified by *P* value significance and are ordered by the protein ratio percentage within that annotation term. The color of each annotation dot is representative of the –log_10_FDR value, and the size corresponds to the number of proteins belonging to the annotation term. (**F**) The Cytoscape stringAPP was used to create protein-protein interaction networks for the significantly elevated proteins in participants with Hi.AI (FC > 2, *P* < 0.05, and Spearman’s *r* > 0.6 with AI). MCODE clustering identified the highly interconnected nodes in the networks. The colors are continuously mapped and increasing FC corresponds to a deeper red color.

Of the 1,317 proteins assayed, 64 proteins were significantly upregulated (fold change [FC] > 2; *P* < 0.05) and 22 were significantly downregulated (FC < 0.05; *P* < 0.05) in patients with LN with high AI (AI ≥ 7) when compared with patients with non–high AI (AI ≤ 6), irrespective of CI scores, as shown in the volcano plot in Figure 1A. Among these 64 upregulated proteins, 57 proteins (referred to as high-AI proteins) exhibited the strongest Spearman correlation with renal pathology AI (*r* > 0.6); all these proteins also exhibited receiver operating characteristic (ROC) AUC values >0.775 when comparing patients with LN with and without high AI. Principal component analysis (PCA) using the high-AI proteins effectively distinguished patients with high AI from those with inactive disease (Figure 1B). Next, these 57 high-AI proteins were subjected to functional pathway enrichment analysis, including Kyoto Encyclopedia of Genes and Genomes (KEGG) pathway analysis and Gene Ontology (GO) analysis. Among the top 10 most-enriched KEGG pathways (Figure 1C), PI3K-AKT signaling, oocyte meiosis, and MAPK signaling were the most prominent, encompassing the largest number of proteins under these annotation terms. The top GO biological processes and molecular functions enriched among the high-AI proteins included signal transduction and regulation, notably MAPK signaling (Figure 1D), protein binding, receptor binding, enzyme binding, and protein domain–specific binding (Figure 1E). Among these proteins, highly interconnected nodes within protein-protein interaction networks were identified using Molecular Complex Detection (MCODE) clustering (Figure 1F), encompassing several important biological pathways.

### Correlation of high-AI proteins with LN pathology and clinical parameters.

**Figure 2:**
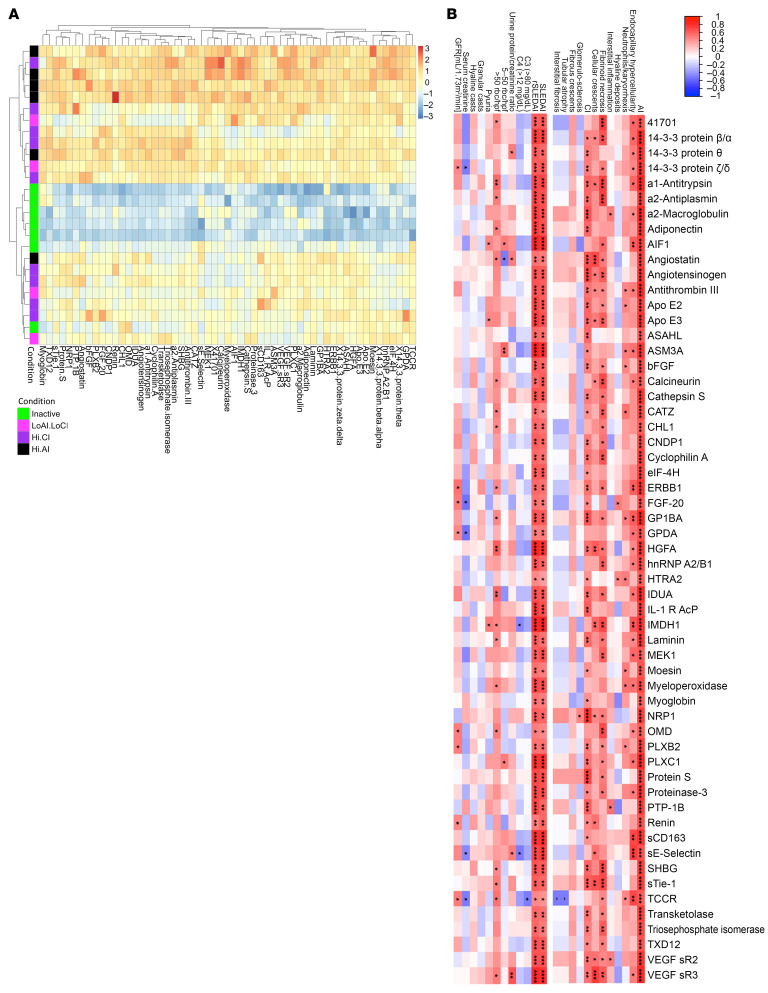
Correlation of high-AI urine proteins with renal pathology and clinical parameters. (**A**) A heatmap representation of the 57 significantly upregulated proteins from the aptamer-based screen elevated in individuals with Hi.AI (*P* < 0.05, Mann-Whitney *U* test; FC > 2; Spearman’s *r* > 0.6 with AI). Proteins are clustered hierarchically. Each row represents a study participant. Each column represents a Cr-normalized protein-level expression. Proteins expressed above the mean are shaded red, those comparable to the mean are shaded yellow, and those below the mean are shaded blue. Subject groupings are color coded as indicated. Note: Two patients had high AI and CI (both were classified under “Hi.AI” in this plot). (**B**) Spearman’s correlation heatmap displaying correlations among the top 57 proteins elevated in participants with Hi.AI and the participants’ clinical metrics as well as their concurrent renal pathology features including CI and its 4 component attributes (glomerulosclerosis, fibrous crescents, tubular atrophy, interstitial fibrosis) and AI and its 6 component attributes (endocapillary hypercellularity, neutrophils/karyorrhexis, hyaline deposits, interstitial inflammation, fibrinoid necrosis, and cellular crescents). *P < 0.05, **P < 0.01, ***P < 0.001, and ****P < 0.0001.

The heatmap in Figure 2A depicts the relative expression profiles of the 57 high-AI proteins in patients with LN (Figure 2A), compared with patients with LN but without high-AI and patients with clinically inactive disease (who exhibited the lowest levels of these 57 proteins).

The correlation of these high-AI proteins with renal histological and clinical parameters was further explored. Although all proteins were significantly correlated with the global AI score, the AI attributes endocapillary hypercellularity, fibrinoid necrosis, and cellular crescents demonstrated the strongest correlation with urine levels of high-AI proteins. However, these proteins did not correlate with renal pathology chronicity attributes such as glomerulosclerosis, fibrous crescents, interstitial fibrosis, or tubular atrophy, as displayed by the heatmap in Figure 2B. Twelve proteins (namely, 41701 [in the 14-3-3 protein family], AIF1, calcineurin, CHL1, GPDA, hnRNP A2/B1, IMDH1, MEK1, myeloperoxidase [MPO], OMD, sE-selectin, and TCCR [aka, IL27RA]) were exclusively correlated with AI but not with CI. Among the clinical parameters, all high-AI proteins correlated positively and significantly with the SLE disease AI (SLEDAI) and the renal SLE disease AI (rSLEDAI), urine RBC count >50 per high-power field was positively correlated with most of the high-AI proteins, whereas circulating levels of C3, C4, and serum creatinine (Cr) had negative correlations, as one might expect. However, the urine protein/Cr ratio was not associated with the majority of these high-AI proteins (Figure 2B).

### Top discriminatory proteins identifying patients with LN with high AI.

**Figure 3:**
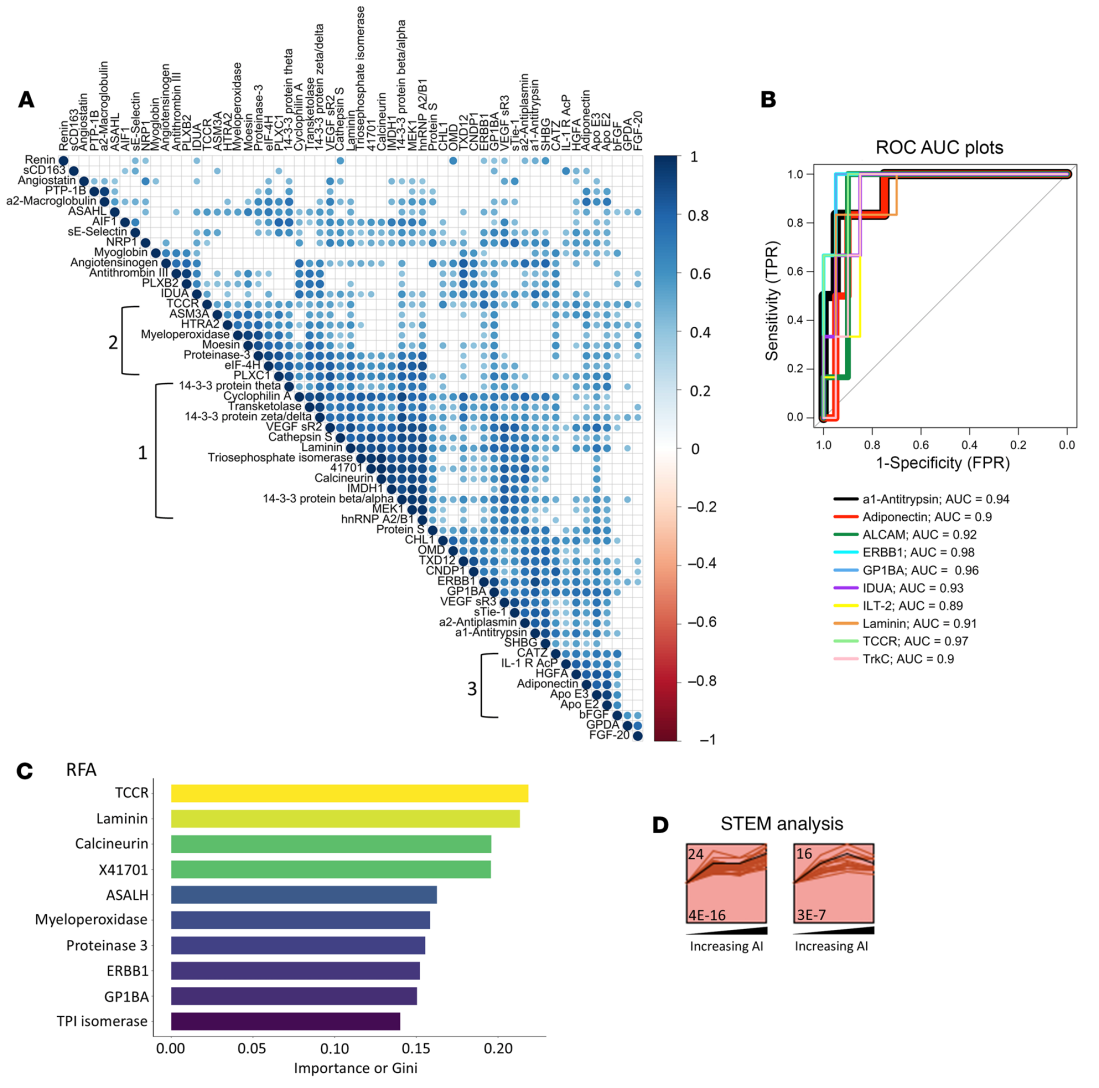
Top discriminatory urine proteins identifying patients with LN with high renal pathology AI. (**A**) The 57 significantly elevated proteins in participants with Hi.AI (FC > 2, *P* < 0.05; Spearman’s *t* > 0.6 with AI) were subjected to protein-protein correlation analysis. Hierarchical clustering was performed. Dark blue corresponds to a positive correlation between protein pairs. The correlation cluster labeled “1” encompasses multiple signaling proteins critical for the activation of immune cells. Cluster 2 represents a neutrophil signature cluster and the cluster labeled “3” encompasses several anti-inflammatory proteins. (**B**) An ROC AUC plot of the top 10 proteins from the aptamer-based screen based solely on ROC AUC values discriminating participants with Hi.AI from all other patients with LN. TPR, true positive rate; FPR, false positive rate. (**C**) RFA shown are the 10 most discriminatory proteins for the identification of participants with Hi.AI. The proteins are ordered by their importance in discrimination, displayed as the Gini coefficient. (**D**) STEM analysis was executed for the top 57 proteins (FC > 2; *P* < 0.05; Spearman’s *t* > 0.6 with AI). Protein expression through increasing AI are plotted. Statistically significant profiles that are similar form a cluster of profiles and are shaded the same color. A total of 10 profiles (each representing a different temporal pattern) were generated by STEM analysis, of which only the statistically significant profiles are displayed. The number in the upper left-hand corner of each box is the number of proteins in each profile. The number in the lower left-hand corner is the *P* value significance of the number of proteins assigned to each profile relative to the expected number, based upon random permutation testing.

The 57 upregulated proteins in patients with LN with high AI were hierarchically organized into several clusters based on expression profiles, using a protein-protein correlation matrix, as shown in Figure 3A. The largest correlation cluster (labeled “1” in Figure 3A) encompassed multiple signaling proteins critical for the activation of immune cells (including calcineurin, cyclophilin A, multiple 14-3-3 family proteins, 41701, MEK1, among others). Also prominent was a neutrophil activation signature cluster (notably, MPO, proteinase 3, moesin, EIF4H, among others; labelled “2” in Figure 3A). Another cluster encompassed anti-inflammatory proteins, including ApoE isoforms, adiponectin, and HGFA (labeled “3” in Figure 3A), whereas the anti-inflammatory macrophage marker CD163 clustered by itself. Multiple endothelial cell activation molecules were also associated with high AI, although they did not cluster together (namely, Tie1, E-selectin, VEGF-R2, VEGF-R3).

We used ROC analysis to identify the top 10 proteins differentiating the patients with LN with high AI from those with low AI. These were ERBB1 (aka, epidermal growth factor receptor [EGFR]), TCCR, GP1BA, α1-antitrypsin, IDUA, ALCAM, laminin, adiponectin, TrkC, and ILT-2, in order of descending AUC values; all had AUC values exceeding 0.89 (Figure 3B). (Three of these proteins, ALCAM, TrkC, and ILT-2, are not included in the top 57 proteins described in Figure 2 and Figure 3A, because their FC values did not exceed 2). The 10 proteins that were most discriminatory for high AI in LN were also identified using an independent machine-learning algorithm, random forest analysis (RFA). This alternative approach identified TCCR, laminin, calcineurin, 41701 (14-3-3 protein family), ASAHL, MPO, proteinase 3, ERBB1, GP1BA, and TPI isomerase as the most discriminatory proteins (Figure 3C). Four proteins — ERBB1, TCCR, GP1BA, and laminin — were identified in common, by both ROC and RFA analysis, as being discriminatory of high renal pathology AI.

To identify urine proteins and functional pathways that progressively increase with increasing AI, short time-series expression miner (STEM) analysis was performed, revealing 2 distinct protein clusters (Figure 3D and Supplemental Data File 1; supplemental material available online with this article; https://doi.org/10.1172/JCI186143DS1). Reactome and KEGG functional pathways enriched among the proteins clustered in the first STEM cluster were related to cell signaling, cell cycle control, and protein translocation. The Reactome pathways implicated by the second STEM cluster focused on communication between different cellular compartments and the proper functioning of various cellular systems. Interestingly, all the cell activation proteins in correlation cluster 1 in Figure 3A and neutrophil activation proteins in correlation cluster 2 in Figure 3A belonged to STEM cluster 1 and progressively increased in expression with worsening renal pathology AI.

### Proteins and pathways enhanced in urine samples of patients with LN with high CI.

**Figure 4:**
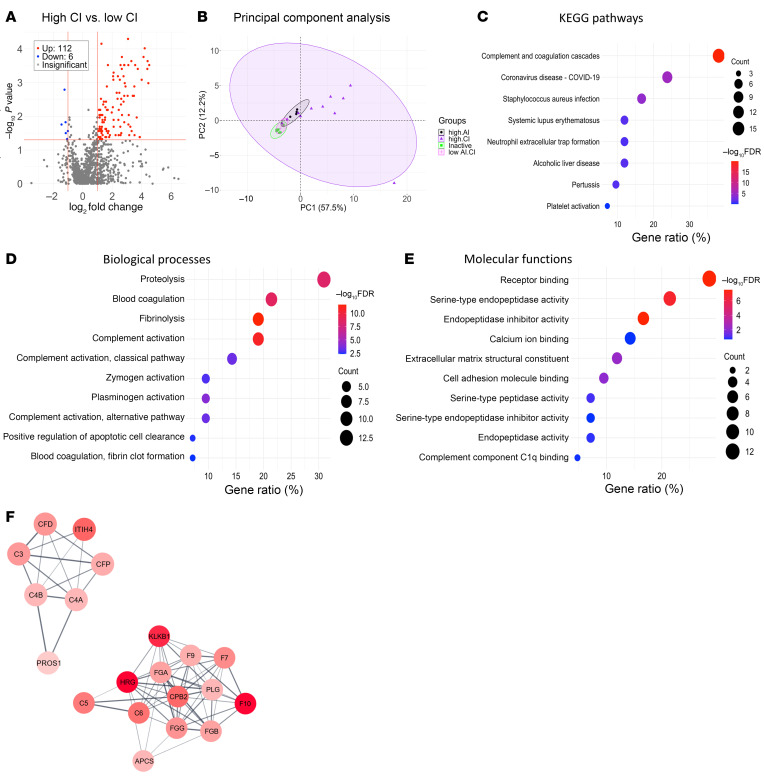
Proteins elevated in urine samples of patients with LN with high renal pathology CI. (**A**) A volcano plot representation of all 1,317 proteins assayed in the aptamer-based screen; log-transformed data were used for analysis. In total, 112 proteins were significantly upregulated with an FC < 0.5 and *P* < 0.05. Of the 112 elevated proteins, 50 had a Spearman’s *r* > 0.6 with concurrent renal pathology CI (here, abbreviated CI). All these proteins had ROC AUC values of greater than 0.8, comparing patients with high CI with other patients with lupus. (**B**) A PCA plot of the 50 significantly elevated proteins in participants with high CI (FC > 2; *P* < 0.05; Spearman’s *r* > 0.6 with CI), with principal components (PCs) displayed on each axis. Concentration ellipses encompass each subject group, color coded as indicated. (**C**–**E**) The 50 proteins from the aptamer-based screen whose levels were elevated in individuals with high CI (FC > 2; *P* < 0.05; Spearman’s *r* > 0.6 with CI) were used for GO and KEGG pathway enrichment analysis. The implicated top 10 KEGG pathways (**C**), biological processes (**D**), and molecular functions (**E**) identified using the Database for Annotation, Visualization and Integrated Discovery are displayed. Annotation details are as listed in Figure 1. (**F**) The Cytoscape stringAPP was used to create protein-protein interaction networks for the significantly elevated proteins in individuals with high CI (FC > 2; *P* < 0.05; Spearman’s *r* > 0.6 with CI). Annotation details are as listed in Figure 1. Reactome pathways implicated include 2 dominant clusters: cluster 1, including formation of fibrin clot (clotting cascade) and hemostasis; and cluster 2, including complement cascade initial triggering of complement, and activation of C3 and C5.

Of the 1,317 proteins assayed, 112 proteins were significantly upregulated (FC > 2; *P* < 0.05) and 6 were significantly downregulated (FC < 0.05; *P* < 0.05) in patients with LN with a high CI (chronicity ≥ 4) compared with patients without a high CI (chronicity ≤ 3), irrespective of their AI scores, as shown in the volcano plot in Figure 4A. The top 50 high-CI proteins were defined as proteins with ROC AUC values exceeding 0.8, with a Spearman’s *r* > 0.6 with the CI score. These proteins effectively distinguished the patients with high CI from others, as revealed by PCA analysis (Figure 4B). Then, these top 50 high-CI proteins were subjected to functional pathway enrichment analysis. Of the KEGG pathways enriched (Figure 4C), complement and coagulation cascades were the most prominent, encompassing the largest number of proteins under these annotation terms. The most enriched GO biological processes included positive regulation of proteolysis, blood coagulation, fibrinolysis, and complement activation (Figure 4D). The top 3 enriched GO molecular functions included receptor binding, serine-type endopeptidase activity, and endopeptidase inhibitor activity (Figure 4E). MCODE clustering identified the highly interconnected nodes in the protein-protein interaction network of the high-CI proteins (Figure 4F) to be centered on hemostasis, fibrin clot formation, and complement cascade activation.

### Correlation of high-CI proteins with LN pathology and clinical parameters.

**Figure 5:**
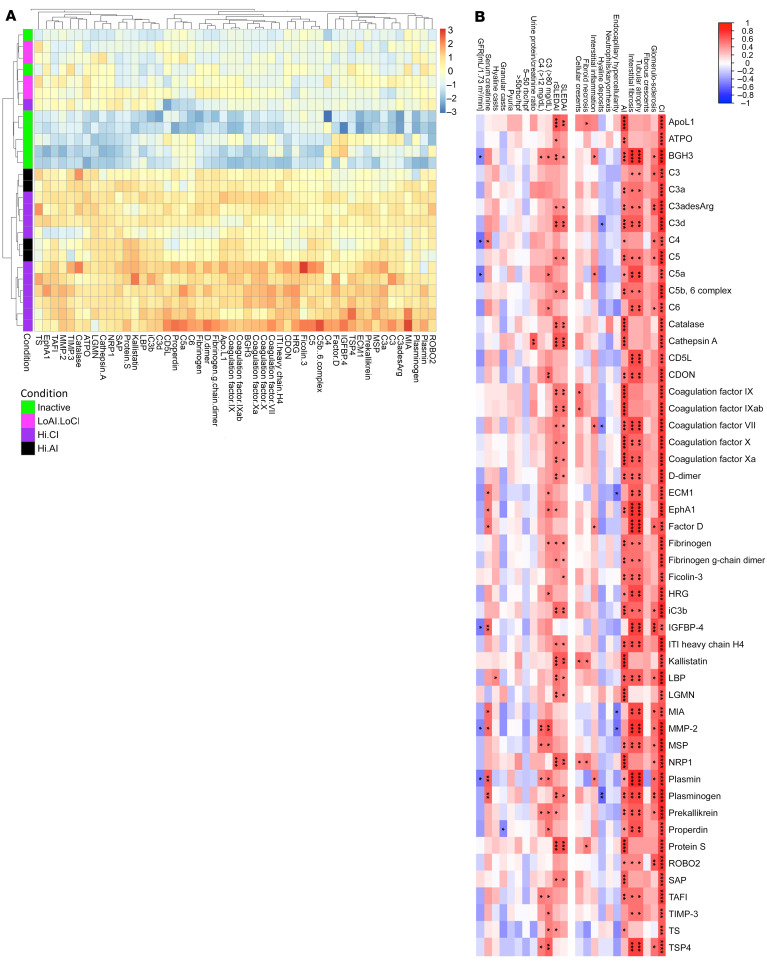
Correlation of high CI urine proteins with LN pathology and clinical parameters. (**A**) A heatmap representation of the 50 significantly upregulated proteins from the aptamer-based screen elevated in individuals with Hi.CI (*P* < 0.05 by Mann-Whitney *U* test; FC > 2; Spearman’s *r* > 0.6 with CI). Annotation details are listed in Figure 2. Note: Two patients had Hi.AI and high CI (Hi.CI); both were classified under “Hi.CI” in this plot. (**B**) A Spearman correlation heatmap displaying correlations among the top 50 proteins elevated in individuals with Hi.CI and the participants’ clinical metrics, as well as their concurrent renal pathology features, as detailed in Figure 2. GFR, glomerular filtration rate. *P < 0.05, **P < 0.01, ***P < 0.001, and ****P < 0.0001.

As depicted in the heatmap in Figure 5A, the top 50 upregulated high-CI proteins correlated significantly with the CI scores, most notably with tubular atrophy and interstitial fibrosis. Though several of the high-CI proteins also correlated with global AI, they did not correlate with endocapillary hypercellularity, fibrinoid necrosis, or other key component features of AI. Urine proteins that were correlated exclusively with CI but not with AI included complement proteins (C3, C6, factor D), ECM turnover proteins (MMP-2, TIMP-3, ECM1, TSP4, MIA) and others, such as IGFBP-4 and AIM/CD5L (Figure 5B).

### Top 10 discriminatory proteins identifying patients with LN with high CI.

**Figure 6:**
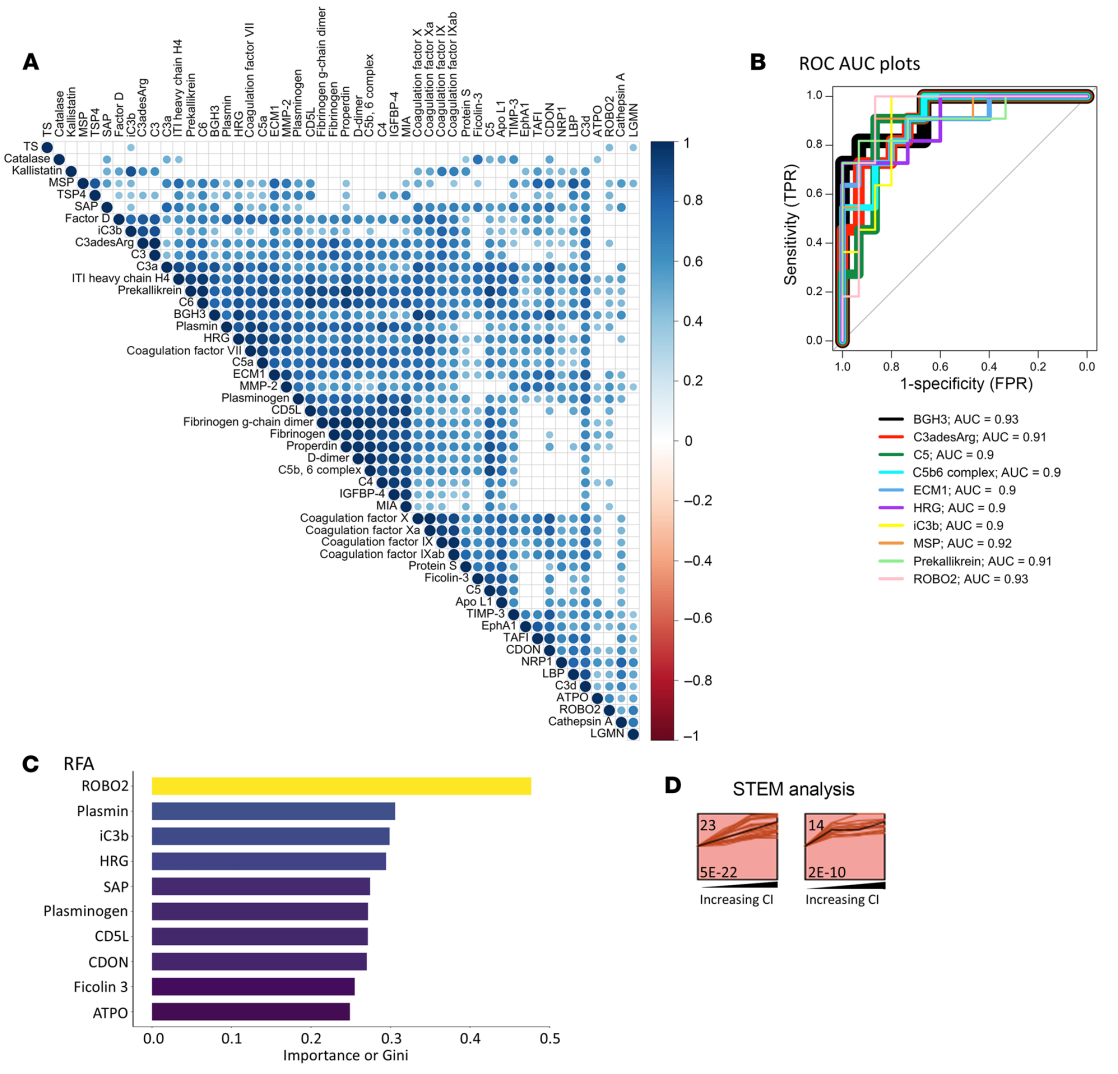
Top discriminatory proteins identifying patients with LN with high renal pathology CI. (**A**) The 50 significantly elevated proteins in participants with a high CI (FC > 2; *P* < 0.05; and Spearman’s *r* > 0.6 with CI) were subjected to protein-protein correlation analysis. Shown is a correlation plot displaying all significant Pearson’s correlations (*P* < 0.05) among these proteins. Hierarchical clustering was performed. Dark blue corresponds to a positive correlation between protein pairs. (**B**) An ROC AUC plot of the top 10 proteins from the aptamer-based screen based on ROC AUC values discriminating participants with a high CI from all other patients with LN. AUC values were calculated using the Delong method with a 95% confidence level. The proteins and their AUC value are differentiated by color. (**C**) The 50 proteins with significantly elevated levels in participants with a high CI (FC > 2; *P* < 0.05; Spearman’s *r* > 0.6 with CI) were subjected to RFA, and the 10 proteins’ most discriminatory of high CI are indicated, ordered by their Gini coefficients. (**D**) STEM analysis was executed for the 50 proteins implicated (FC > 2; *P* < 0.05; Spearman *r* > 0.6 with CI) to identify urine proteins that increase progressively with worsening CI scores. Annotation details are as listed in Figure 3. A total of 8 profiles or clusters (each representing a different temporal pattern) were generated by STEM analysis, of which only the statistically significant clusters are displayed.

When clustered based on expression, the 50 upregulated high-CI proteins were hierarchically organized into several inter-related clusters, most intermixed with complement and coagulation proteins (Figure 6A). By ROC analysis the top 10 proteins differentiating patients with LN with high CI from those without were BGH3, ROBO2, MSP, C3adesArg, prekallikrein, C5, C5b6 complex, ECM1, HRG, and iC3b, in order of descending AUC values with the minimal AUC value being 0.90 (Figure 6B). The most discriminatory proteins for high CI in LN were also identified using an independent machine-learning algorithm, RFA. This alternative approach identified ROBO2, plasmin, iC3b, HRG, SAP, plasminogen, CD5L, CDON, ficolin 3, and ATPO, ordered by their importance in discrimination represented by the Gini coefficient (Figure 6C). To identify urine proteins and functional pathways that progressively increased with CI, STEM analysis was performed, revealing 8 clusters, each representing a different pattern, the largest 2 of which are displayed (Figure 6D). Functional pathways represented by the proteins in the top 2 clusters were composed almost exclusively of complement and coagulation cascade proteins. Thus, increasing levels of urine complement and coagulation proteins reflect progressively worsening renal pathology CI scores in LN.

### Complement and coagulation proteins associated with LN pathology.

**Figure 7:**
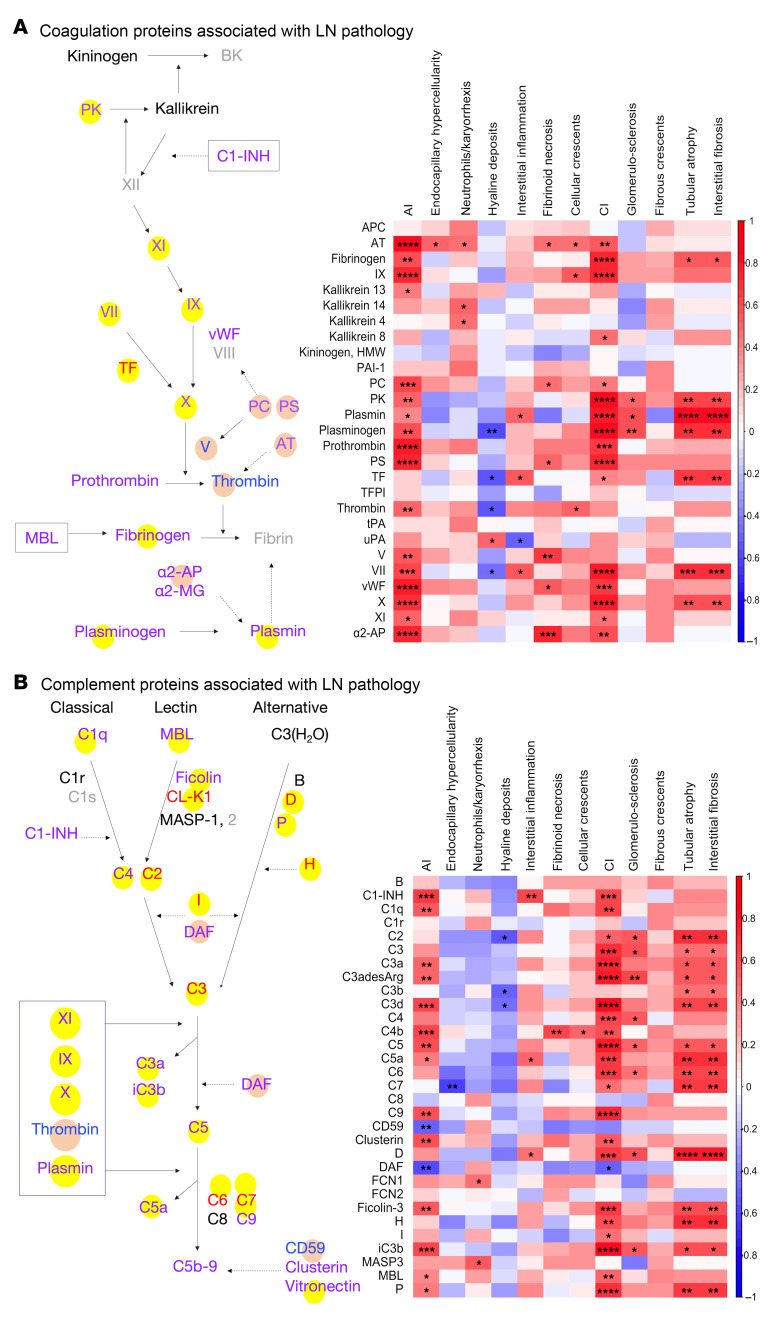
Coagulation and complement proteins associated with LN pathology. (**A**) The blood coagulation cascade, highlighting molecules whose levels in urine are significantly correlated with renal pathology AI (blue font), CI (CI) (red font), or both (purple font) in LN. Other proteins are listed in black font (interrogated but not significantly changed) or grey font (not interrogated by the proteomic screen). Uninterrupted arrows indicate activation, and interrupted arrows signify inhibition or cleavage of downstream protein or substrate. The yellow bubbles highlight only proteins significantly elevated in LN with high-CI versus low-CI (FC ≥ 2; *P* < 0.05) or higher in CI than in AI by at least 10%. The pink bubbles highlight proteins whose levels were significantly elevated only in patients with LN with high-AI versus low-AI (FC ≥ 2; *P* < 0.05) or higher in AI than in CI by at least 10% (in terms of correlation coefficient or FC). Also shown is a Spearman correlation heatmap displaying correlations among the 27 coagulation-related proteins and renal pathology metrics. *P < 0.05, **P < 0.01, ***P < 0.001, and ****P < 0.0001. (**B**) The complement activation pathway highlighting molecules whose levels in urine are significantly elevated with AI, CI, or both. See **A** for other annotation details. Also shown is a Spearman correlation heatmap displaying correlations among the 32 complement related proteins and their paired renal pathology metrics, as detailed in **A**. α2-AP, MG, α2-antiplasmin, α2-macroglobulin; APC, activated protein C; AT, antithrombin; B, factor B; BK, bradykinin; C1 INH, C1 esterase inhibitor; C4BP, C4 binding protein; CL-K1, collectin kidney 1; D, factor D; DAF, decay-accelerating factor; FDP, fibrin degradation products; H, factor H; I, factor I; MAP-1, MBL/ficolin-associated protein 1; MASP, mannan-binding lectin-associated serine protease; MBL, mannose-binding lectin; MCP, membrane cofactor protein; P, properdin; PK, prekallikrein; sMAP, small MBL-associated protein; TM, thrombomodulin; tPA, tissue plasminogen activator; uPA, urokinase.

As the above analysis pointed to the coagulation cascade and the complement cascade, proteins in these 2 pathways were further interrogated systematically. Twenty-seven coagulation cascade proteins interrogated by the aptamer-based screen were subjected to correlation analysis with the renal pathology AI score, CI score, and their component attributes captured from the same patients, as displayed in Figure 7A. Kallikrein 13, thrombin, and factor V were significantly and exclusively correlated with AI, whereas kallikrein 8 and tissue factor (TF) were significantly and exclusively correlated with CI. The remaining 14 proteins, including AT, fibrinogen, factor IX, PC, prekallikrein, plasmin, plasminogen, prothrombin, PS, factor VII, vWF, factor X, factor XI, and α2-AP, were significantly correlated with both AI and CI. Most of the proteins correlated with CI were also significantly correlated with 2 specific CI attributes: tubular atrophy and interstitial fibrosis. Similarly, 32 complement proteins interrogated by the aptamer-based screen were also evaluated for their correlation with renal histological AI and CI scores (Figure 7B). Although most complement proteins were significantly positively correlated with CI and specific CI attributes, correlation with AI attributes was modest or negative.

### Complements activate profibrotic pathways in macrophages and tubular cells.

**Figure 8:**
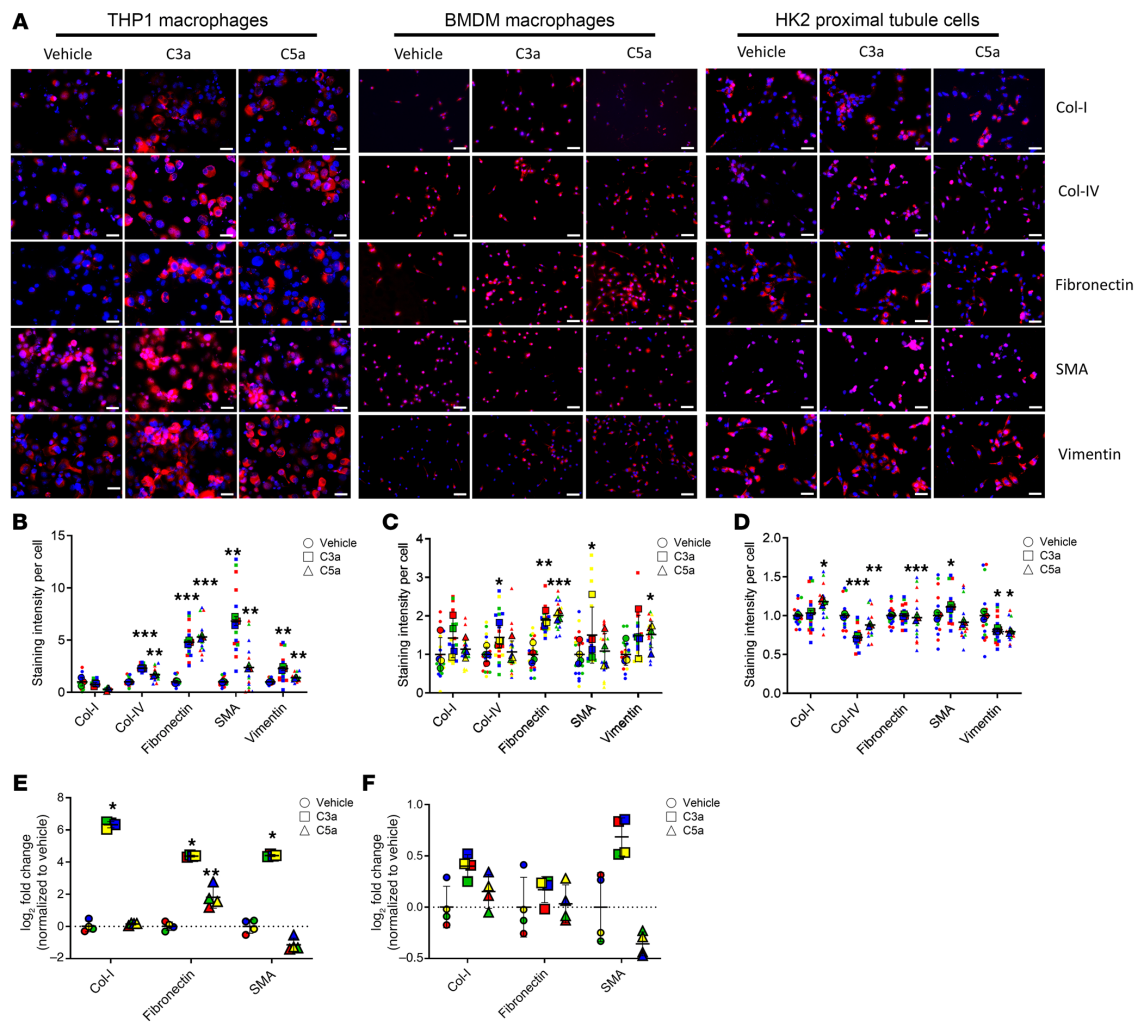
Complement C3a and C5a trigger the expression of ECM proteins in macrophages and proximal tubule cells. (**A**) Shown are representative fields from 3 independent experiments. Complement proteins C3a and C5a increased the expression of ECM proteins in THP1 macrophages, BMDMs, and HK2 proximal tubule cells after 72 hours of treatment in serum-free medium. Scale bars: 50 μm. (**B**–**D**) The scatter plots show the mean staining intensity per THP-1 macrophage (**B**), BMDM (**C**), and HK2 proximal tubule cell (**D**), normalized to expression levels in their respective vehicle-treated groups. Each data point corresponds to quantified fluorescence intensity in a single field of view (FOV) from the microscope, and the larger dots represent the average of FOVs in biological replicates, each of which is color coded. RT-qPCR analysis of ECM protein coding genes were measured in BMDMs (**E**) and in HK2 proximal tubule cells (**F**). Gene expression was normalized to the expression of 18S ribosomal RNA in the same sample and then normalized to the expression level of vehicle-treated group (*n* = 4). *P < 0.05, **P < 0.01, and ***P < 0.001 by 2-tailed Student’s *t* test.

Given the prominent association or correlation of high CI with complement proteins and ECM turnover, we next explored if these were mechanistically linked. When added in vitro, complement proteins C3a and C5a increased the expression of several ECM proteins in THP-1 macrophages, bone-marrow-derived macrophages (BMDMs), and HK2 proximal tubule cells (Figure 8A), as quantified in Figure 8, B–D, respectively. These findings were validated at the RNA level by RT-qPCR in BMDMs (Figure 8E) and in HK2 proximal tubule cells (Figure 8F), with C3a being more potent.

## Discussion

Based on an unbiased, comprehensive, aptamer-based proteomic screen, this study has identified urinary molecular signatures associated with renal pathology activity or chronicity changes in LN. Urinary readouts of endocapillary hypercellularity, fibrinoid necrosis, and cellular crescents (i.e., high renal pathology AI) include leukocyte/podocyte activation/cell signaling (e.g., PI3K/Akt, MAPK, ERK, calcineurin, cyclophilin A, multiple 14-3-3 family proteins, 41701, MEK1), neutrophil activation (e.g., MPO, proteinase 3, moesin, EIF4H), endothelial activation (e.g., Tie1, E-selectin, VEGF-R2, VEGF-R3,), and markers of inflammation or anti-inflammation (e.g., ApoE isoforms, adiponectin, HGFA), including the M2 macrophage marker CD163. Of importance, several of these proteins associated with high AI in LN have been independently validated by a recent proteomic screen using an Ab-based array, where urinary cyclophilin, M2 macrophage markers (CD163, CD206), and neutrophil markers (proteinase-3) were noted to be indicative of high AI (18). The implicated pathways are all of pathogenic and therapeutic significance in LN. Indeed, the calcium signaling pathway (involving calcineurin, cyclophilin A, and multiple 14-3-3 family proteins) is central not only to leukocyte signaling but also to podocyte survival (19, 20). The PI3K/Akt/mTOR pathway is activated in murine LN and is downregulated by rapamycin (21). Similarly, MAPK and ERK pathways have also been implicated in LN pathogenesis (22). This pathway is the target of drugs that are effective in LN, including rapamycin, and the FDA-approved drug, voclosporin (23). We posit that urinary levels of these proteins (e.g., calcineurin, cyclophilin A, 14-3-3 family proteins) may serve as useful indicators of clinical remission in LN, where renal pathology AI is also reduced, particularly in patients treated with calcineurin inhibitors.

Likewise, neutrophil signatures as well as neutrophil extracellular traps have been implicated in LN (24). Blockade of netosis ameliorates LN, though not in all animal models (25). M2 macrophages and the M2 macrophage marker CD163 have been implicated in LN in several studies and are associated with renal pathology AI (15, 26). RFA identified TCCR/WSX-1, which is the IL-27 receptor subunit α, as the urinary protein most discriminatory for high AI. WSX-1 is highly expressed on CD4^+^ T cells, NK cells, NKT cells, and macrophages. IL27R/WSX-1 plays an inhibitory role by regulating cell activation and cytokine production. MRL/lpr mice with WSX-1 overexpression exhibited a prolonged lifespan with no apparent development of nephritis (27). ROC AUC analysis identified ERBB1 as the urinary protein most discriminatory for high AI. EGFR is a tyrosine kinase transmembrane receptor that plays important roles in cell proliferation, survival, and differentiation. Recently, we have confirmed that EGFR is significantly elevated in proliferative LN kidneys (28). Finally, other proteins previously reported to be associated with clinical or histological activity in LN, such as ALCAM and E-selectin, were revalidated as urinary markers of high renal pathology activity in this study (13, 29).

On the other hand, complement and coagulation cascade proteins, as well as ECM turnover proteins (MMP-2, TIMP-3, ECM1, TSP4, MIA) emerged as the strongest readouts of concurrent tubular atrophy and interstitial fibrosis (i.e., high CI) in LN. The participation of complement in LN pathogenesis is widely accepted. Immune complexes from circulation or formed in situ by the binding of circulating autoantibody to intrarenal antigens activate the complement cascade, forming the membrane attack complex (MAC), leading to the production of proinflammatory mediators, recruitment of immune cells, and eventually, tissue damage (30). The intensity of immune complex deposition in the tubular basement membrane, strongly positive for complements C3d, C1q, and C4d, correlates positively with renal pathological indices (31). Although the activation of the classical pathway has long been accepted as the main source of complement activation in LN, the lectin pathway and the alternative pathway are also implicated in glomerular injury (32, 33).

Patients with tubular basement membrane C4d staining have higher disease activity as measured by SLEDAI and higher AI and CI (34). C4d along peritubular capillaries is significantly correlated with the CI in LN (35). The formation of the C5b-9 MAC promotes the progression of chronic interstitial damage and renal failure (36). Tubular MAC deposition is associated with tubular atrophy, interstitial fibrosis, glomerular sclerosis, and global CI scores, and glomerular MAC deposition is associated with hypertension and worse treatment response (37, 38). Patients with LN with thrombotic microangiopathy have stronger staining intensity and deposition of MBL, MASP1/3, CFB, CFD, C4d, and VWF. Indeed, renal pathology CI and interstitial fibrosis are worse in patients with LN with thrombotic microangiopathy involving the lectin and alternative pathways (39). Importantly, intrarenal complement gene expression (C1R, C1QB, C6, C9, C5, MASP2) predicts nonresponse to induction therapy (40), alluding to the potential pathogenic role of intrarenal complement gene expression in chronic kidney disease. In resonance with the reported findings, urinary C3 and C9, as measured by an orthogonal proteomic platform, have been shown to reflect tubular atrophy and interstitial fibrosis in LN and to correlate with key mediators of kidney fibrosis, including TGFβR1, PDGFβ, and PDGFRβ (41).

**Figure 9:**
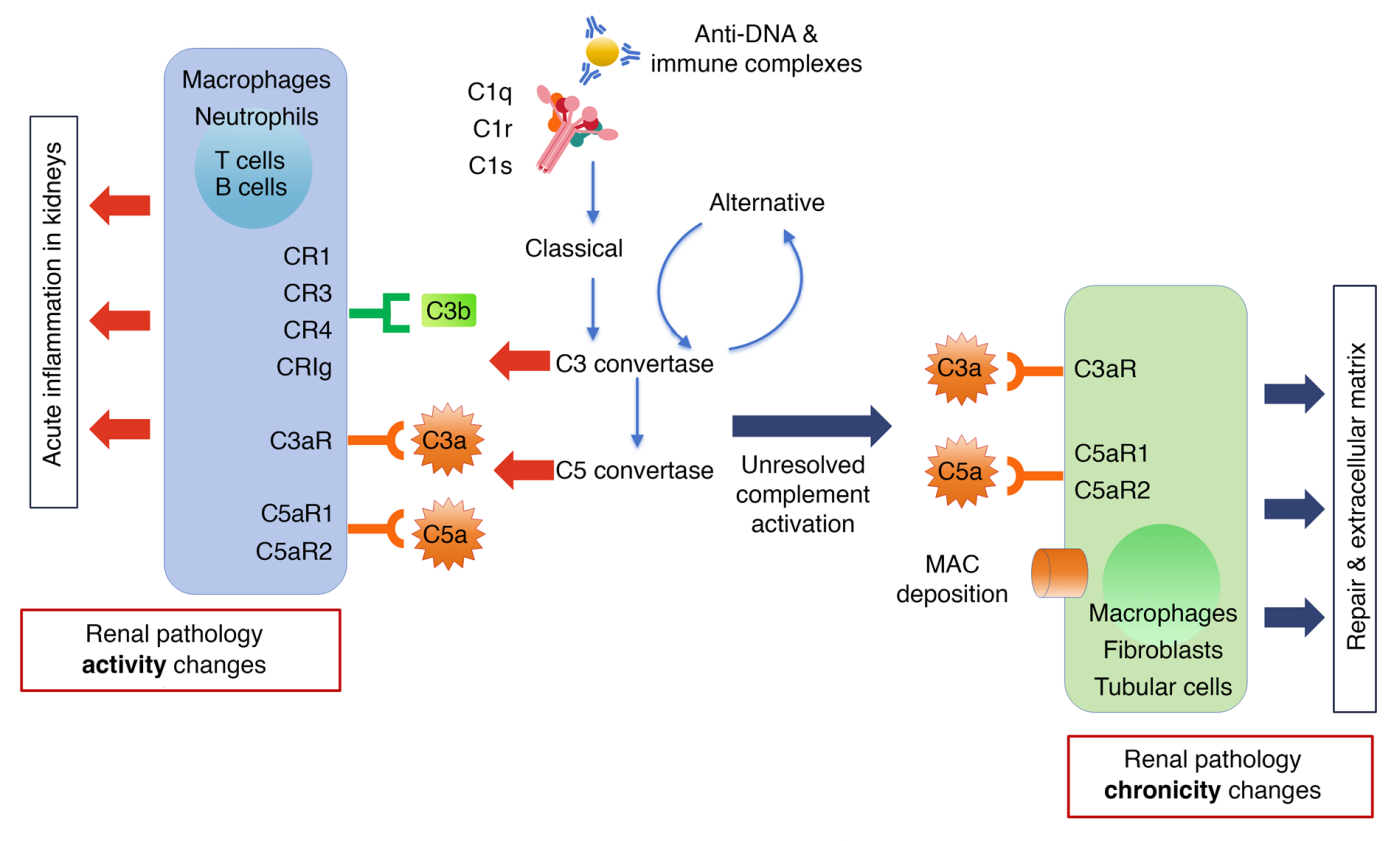
The biphasic role of complement proteins in driving renal pathology activity and chronicity in LN. Circulating immune complexes and Abs planted directly within glomerular and tubulo-interstitial regions of the kidneys may fix complement, resulting in complement activation. The alternative pathway may further amplify complement activation within the kidneys. The products of C3 and C5 convertases, including the anaphylatoxins C3a and C5a, engage cognate receptors on a wide spectrum of immune cells, leading to immune cell activation, release of cytokines and chemokines, and acute inflammation, leading to high AI, as depicted on the left. Long-standing, unresolved complement activation and eventual formation of MAC may additionally engage and activate more immune and renal-resident cells, leading to tissue damage and repair, ECM deposition, and renal fibrosis, leading to high CI, as depicted on the right.

Our findings that C3a and C5a can promote profibrotic changes in renal resident cells and infiltrating leukocytes resonate well with similar observations in the literature. C5a stimulates proliferation and activation of renal fibroblasts, and expression of IL-1α, IL-6, and TGF-α in renal tubular epithelial cells and monocytes and macrophages. Deficiency of C5aR1 protects mice from the development of fibrosis by attenuating deposition of ECM components, diminishing cellular infiltrates of leucocytes and macrophages, and reducing proinflammatory and profibrogenic mediators in the kidney (42). The C5aR1 antagonist PMX53 significantly reduces renal injury and tubulointerstitial fibrosis (42). Similarly, in LN, renal expression of C5aR mRNA and protein is significantly increased in MRL/lpr mice, whereas blockade of C5aR delays death and renal disease in these mice (43). Synthesizing all the above lines of evidence, we propose a 2-phase model whereby complement may initially drive activity changes in LN but eventually leads to renal pathology chronicity, as described in Figure 9.

The coagulation cascade has also been implicated in the pathogenesis of LN. Coagulation factors reciprocally influence immune responses by activating immune cells, as reviewed by Wilhelm et al. (44). Glomerular deposition of cross-linked fibrin has been documented in LN (45), and we have reported the biomarker potential of urinary prothrombotic molecules (i.e., TF), antithrombotic molecules (i.e., tissue factor pathway inhibitor [TFPI]), and fibrinolytic molecules (plasmin and d-dimer) in LN (46). Bayesian network analysis also revealed that urine plasmin was closely related with the CI in LN and negatively correlated with eGFR (46). In the present study, we confirmed that both urine plasmin and D-dimer were significantly correlated with renal pathology indices (data not shown), particularly with the CI. Likewise, the expression of plasminogen activator inhibitor-1 (PAI-1) is increased in renal tissue in MRL/lpr murine LN, reflecting an imbalance between coagulation and fibrinolysis (47). The increase in PAI-1 and TF, and the decrease in u-PA, in MRL/lpr kidneys may promote the formation of microthrombi and thus contribute to the progression of LN (48).

The complement and coagulation pathways, evolutionarily related enzymatic cascades, are inextricably intertwined in their activation mechanisms and roles in the thromboinflammatory response to injury and infection (49, 50). Complement activation products and complement regulators both cross-modulate blood coagulation. For example, the MASP and MAC activate and promote coagulation; C5a induces endothelial TF and PAI-1 expression; C4b-binding protein affects the level of free protein S (which binds to protein S and thus affects natural anticoagulation), and the C1-inhibitor inhibits the activity of fXIIa, fXIa, and kallikrein in the coagulation system. Conversely, coagulation proteins clearly cross-regulate complement activation: factor Xa, plasmin, thrombin, and kallikrein can act as C3 convertases; factor XII and kallikrein activate complements; von Willebrand factor binds complements and the inhibitors; and TFPI downregulates complements (50–52). Moreover, the coagulation cascade and complement system can also interact with each other indirectly through the regulation of inflammatory mediators, as has been studied in the context of SLE disease severity (53).

Finally, it should not be surprising that urinary proteins associated with ECM turnover (namely, MMP-2, TIMP-3, ECM1, TSP4, and MIA) emerged as the strongest readouts of concurrent tubular atrophy and interstitial fibrosis (i.e., high CI) in LN, given the direct role of these molecules in renal fibrosis. In particular, enzymes that degrade the ECM (MMPs) and the proteins that inhibit these enzymes (TIMPs) have already been ascribed a central role in renal fibrosis (54). Urinary levels of these proteins may serve as noninvasive harbingers of renal fibrosis in LN. Assembly and use of carefully fabricated panels of urinary proteins that are indicative of high renal pathology AI and/or CI may help monitor the status of renal pathology after induction therapy in LN, in a noninvasive manner, without the need for repeat renal biopsies. The recent advent of smartphone-readable, point-of-care, vertical flow rapid tests for assaying urinary biomarkers in LN may eventually pave the way toward home-based self-monitoring of renal status (55).

Limitations of the study include the small sample size. Because the high-AI group included patients with low and high CI, and because the high-CI group included patients with varying AI values, futures studies should incorporate all 4 possible disease groups of LN (low for both AI and CI, high for both AI and CI, as well as patients discordant for these 2 indices) to allow for a more granular analysis. The proteins uncovered that are correlated with LN histological AI or CI need to be further validated in independent cohorts, and their ability to predict AI or CI should be confirmed in longitudinal follow-up studies for which protocol kidney biopsy specimens are available. On the other hand, several of the identified proteins have previously been independently reported, using orthogonal proteomic platforms, hence substantiating the validity of the identified biomarkers (18, 41). Finally, further mechanistic studies are warranted to delineate the precise cells within LN kidneys wherein the coagulation and complement factors operate to drive activity changes initially, and chronicity changes eventually in LN.

## Methods

### Sex as a biological variable.

Our study examined male and female human study participants.

### Patients and samples.

Archived urine samples from patients seen at the University of Texas Southwestern Medical Center Renal Clinic, Dallas, TX, were used in this study. Patient demographics and pathology attributes are summarized in Table 1. All urine samples were from patients with LN undergoing their first renal biopsy, before commencement of immunosuppressive treatment. Pediatric patients were excluded from this study. Clean-catch midstream urine samples were collected in sterile containers and either placed on ice or refrigerated within 1 hour of sample collection. The samples were then aliquoted and stored at –80°C. At each sample collection visit, the patients were assessed by the attending physician, and the following data were obtained: SLEDAI, rSLEDAI, physician global assessment, complete blood cell count; erythrocyte sedimentation rate; Cr, complement C3 and C4, and anti-dsDNA levels; urinalysis; urine protein/Cr ratio; histological AI with its component attributes; and CI with its component attributes. For all patients, the hybrid SLEDAI was used, whereby proteinuria was scored if it was >0.5 g/24 h. The rSLEDAI sums the renal components of the SLEDAI, including hematuria (>5 RBCs/high-power field), pyuria (>5 WBCs/high-power field), proteinuria (>0.5 g/24 h) and urinary casts. Active LN was defined as biopsy-proven LN if rSLEDAI was >0. In this study, high AI was defined as AI score ≥7, and low AI as a score of 0 to 6; high CI was defined as CI score ≥4, and low CI as a score of 0 to 3. Inactive LN was defined as rSLEDAI = 0.

### Aptamer-based screening of LN urine.

Urine samples obtained at the time of renal biopsy from 26 patients with lupus were centrifuged and subjected to an aptamer-based screen (SOMAscan; Standard BioTools) of 1,317 distinct human proteins, following protocols detailed previously (13). Briefly, all urine samples were clarified by centrifugation before use and subjected to aptamer-based screening. This assay uses aptamer-protein interactions to detect proteins within a sample. In the assay, aptamer-coated streptavidin beads first were added to the sample to allow the aptamers to bind to the proteins. Next, the bound proteins were biotinylated, and the aptamer-protein complexes were cleaved off the streptavidin beads. These aptamer-protein complexes were then conjugated to a second streptavidin bead, and aptamers were separated from the proteins and quantitated by hybridization to a DNA microarray. Relative fluorescence units for each protein were normalized to urinary Cr and statistically analyzed. The median limit of detection (LOD) of the aptamer based screen is 1.6 pg/mL. The LOD was determined by spiking proteins into buffer prior to the assay. The limits of quantitation (LOQ) were established along with the LOD, and the median lower LOQ value is approximately 3-fold higher than the LOD.

### Cell culture.

THP1 human monocyte cells (ATCC TIB-202) were cultured in RPMI plus 10% FBS + 1% penicillin-streptomycin (Pen-Strep). THP1 suspension cells were seeded into treatment plates and 100 ng/mL PMA) was added to differentiate them to adherent macrophages. After 3 days of differentiation, the medium was replaced with serum-free medium for 24 hours, after which the cells were treated for 72 hours with either vehicle or 10 ng/mL C3a (R&D Systems 3677-C3-025) or 10 ng/mL of C5a (R&D Systems 2037-C5-025/CF). BMDMs were generated using the isolated BM from the femur of C57/B6 mice (Jax 000664) and cultured in RPMI plus 10% FBS plus 1% Pen-Strep plus 10 ng/mL M-CSF (Biolegend 576406) for 5 days. Medium was then replaced with serum-free medium for 24 hours, after which the cells were treated with either vehicle or C3a (10 ng/mL) or C5a (10 ng/mL) for 72 hours. HK-2 proximal tubular cells (ATCC CRL-2190) were cultured in keratinocyte serum-free medium (Life Technologies 17005-042) with 5% FBS. After seeding into the treatment plates and reaching 70% confluence, the medium was replaced with serum-free medium for 24 hours. The cells were then treated with either vehicle or C3a (10 ng/mL) or C5a(10 ng/mL) for 72 hours.

### cDNA synthesis and RT-qPCR.

RNA was extracted from cells by lysing them in TRIzol reagent (Invitrogen 15596026) and following manufacturer’s instructions. The extracted RNA was quantified and 2 μg of RNA was used to synthesize cDNA using a cDNA reverse transcription kit (Applied Biosystems 4368814). Quantification of gene expression by RT-qPCR was performed in the Applied Biosystems Cycler using the iQ SYBR Green Supermix (Bio-Rad 1708882). 18S ribosomal RNA was used as a normalization control, and the relative expression levels of transcripts were calculated by the comparative Ct method.

### Immunocytochemistry.

Cells on coverslips were fixed with 4% paraformaldehyde for 30 minutes on ice, after which membrane permeabilization was performed using 0.1% Triton X-100. The coverslips were incubated in blocking buffer (5% BSA and 5% normal goat serum) for 1 hour, after which primary Abs against collagen I (Abcam ab34710), collagen IV (Invitrogen PA5-104508), fibronectin (Invitrogen PA5-29578), SMA (Abcam ab7817), and vimentin (Abcam ab20346), diluted in blocking buffer, were added and incubated overnight. Goat anti–rabbit secondary Ab (Jackson ImmunoResearch 111-585-003) or goat anti–mouse secondary Ab (Jackson ImmunoResearch 115-545-146) diluted in blocking buffer was then applied on the coverslips and incubated for 1 hour. After washing the unbound Abs, the coverslips were mounted on a slide with DAPI-Fluoromount-G (SouthernBiotech 0100-20). ImageJ software was used to extract the signal intensity from each field of view and enumerate cells based on DAPI staining. The staining intensity per cell was normalized to vehicle-treated group and then compared across groups.

### Statistics.

Data were plotted and analyzed using Prism, version 7 (GraphPad), Microsoft Excel, or R. All data in this study were analyzed using the Mann-Whitney *U* test, because several data sets were not normally distributed or two-tailed Student’s *t* test for experiments with cell cultures. Likewise, the Spearman and Pearson methods were used for correlation analysis. Sensitivity, specificity, AUC ROC, positive predictive value, and negative predictive value were calculated using easyROC software or R. Data from each group were imported into R for cluster analysis and heatmap generation. For clustering, proteins were clustered in an unsupervised manner based on Euclidean distance. R was then used to generate a volcano plot of log_2_ FC of expression versus the –log_10_ *P* value, as determined by Mann-Whitney *U* test. The volcano, PCA, and correlation plots were created in R using the *readr*, *readxl*, *gplots*, *ggplot2*, *ggplot.multistats*, *scatterplot3d*, *Hmisc*, *data.table*, and *corrplot* packages. Random forest classification analysis, a machine-learning algorithm for dimensionality reduction, was executed using R, using the Gini index for comparing biomarkers. GO and KEGG functional enrichment analyses were completed using the Database for Annotation, Visualization and Integrated Discovery, version 6.8. The top biological processes, molecular functions, and KEGG pathways were plotted using R. The packages used included *readxl* and *ggplot2*. The size of the dots represents the count/hit number of genes belonging to the annotation term, and the color of the dots represent –log_10_FDR value.

### Study approval.

All patients gave informed consent, and this study was approved by the IRB of UT Southwestern Medical Center, Dallas, TX, and the University of Houston, Houston, TX, under the IRB approval no. 14074-01.

### Data availability.

The primary data are available from the corresponding author upon reasonable request. The raw data used to generate the figures are available in the Supporting Data Values file.

## Author contributions

CM designed the study and revised the manuscript. TZ performed the experiments, analyzed the data, and wrote the original draft. JC analyzed the data and reviewed the manuscript. ASCLST performed the cell experiment and reviewed the manuscript. KV, VS, and SK performed the experiments, and reviewed the manuscript. RS collected the samples and reviewed the manuscript.

## Supplementary Material

Supplemental data set 1

Supporting data values

## Figures and Tables

**Table 1:**
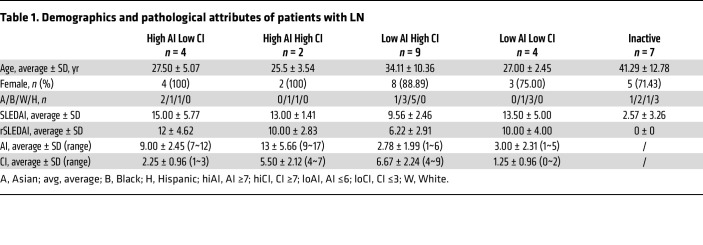
Demographics and pathological attributes of patients with LN

## Version 1 10/01/2025

Electronic publication

## Version 2 01/28/2026

Added conflict of interest statement
